# Supplementary material for: Computer modelling of connectivity change suggests epileptogenesis mechanisms in idiopathic generalised epilepsy
Source: Neuroimage Clin. 2019 Jan 11;21:101655. doi: 10.1016/j.nicl.2019.101655 (PMC6356007; doi:10.1016/j.nicl.2019.101655)
Supplement: Supplementary file 1 — SSupplementary material [file mmc1.pdf]

# Supplementary information: Computer modelling of connectivity change suggests epileptogenesis mechanisms in idiopathic generalised epilepsy


Nishant Sinha[a,b], Yujiang Wang[a,b,c], Justin Dauwels[d], Marcus Kaiser[a,b], Thomas Thesen[e,f], Rob Forsyth[a], Peter Neal Taylor[a,b,c]

[a]*Institute of Neuroscience, Faculty of Medical Sciences, Newcastle University, Newcastle upon Tyne, UK*
[b]*ICOS, School of Computing, Newcastle University, UK*
[c]*Institute of Neurology, University College London, UK*
[d]*School of Electrical and Electronic Engineering, Nanyang Technological University, Singapore*
[e]*Department of Neurology, School of Medicine, New York University, NY, USA*
[f]*Department of Physiology & Neuroscience, St. Georges University, Grenada, West Indies*


## Contents


**1 Supplementary Figures** **1**
- 1.1 Connectometry analysis: Seeding pre-defined regions of interest . . . . . . . . . . . . . 1
- 1.2 Connectometry analysis: Seeding whole brain . . . . . . . . . . . . . . . . . . . . . . . . . 5
- 1.3 Thalamo-frontal white-matter integrity is preserved in IGE patients . . . . . . . . . . 6
- 1.4 Bifurcation diagram illustrating detailed model dynamics . . . . . . . . . . . . . . . . . . 7
- 1.5 Whole-tract approach obscures significant local differences in white matter . . . . . . . 8

**2 Supplementary Tables** **9**
- 2.1 Information on control subjects . . . . . . . . . . . . . . . . . . . . . . . . . . . . . . . . 9
- 2.2 Values of the parameters incorporated in the model. . . . . . . . . . . . . . . . . . . . . 9


## 1. Supplementary Figures

### *1.1. Connectometry analysis: Seeding pre-defined regions of interest*

In this section, we extend our connectometry results in section 3.2 to all the pre-defined regions of interest (ROIs) in the JHU white matter atlas excluding the ROIs in cerebellum, brain-stem, and corpus callosum. The corpus callosum defined in the JHU atlas overlaps considerably with other ROIs, therefore, we instead chose corpus callosum ROIs from the freesurfer Destrieux atlas. Additionally, thalamus-proper ROI was chosen from the Destrieux atlas because it is not defined in the JHU white-matter atlas.

Connectometry analysis, as described in section 2.3.2, was repeated for each ROI set as seed. The pipeline followed is the same as described in Figure 1 of the manuscript. All parameters and threshold used are also the same as described in Figure 1 and section 2.3.2. Results for each ROI are shown in Supplementary Figure 1 (presented in three parts on pg 2-4). Tracts with significantly reduced (increased) gFA in IGE patients are plotted for an exemplary threshold value depicted in red (blue) star in Supplementary Figure 1.

As shown in Supplementary Figure 1 panel (a) to (i), we found that only the cortico-cortical tracts have significantly reduced gFA in IGE patients compared to controls. Additionally, a significant increase in gFA was detected for the cortico-thalamic tracts illustrated in Supplementary Figure 1 panel (h) to (j). In panel (h) and (i), both significant increase and decrease were detected for the seed ROI shown. However, the tracts showing decrease are the cortico-cortical tracts and increases are the cortico-thalamic tracts emanating via the seeded ROI. Seeding only the thalamic ROIs from the Destrieux atlas (Supplementary Figure 1 panel (k)) makes it apparent that the cortico-thalamic tracts are increased in IGE patients. Note that in panel (k), in addition to the superior-inferior cortico-thalamic tracts shown in blue, we also detected a few additional tracts shown in red. These are the fornix tracts which are detected due to the partial overlap between the thalamic ROI defined in Destrieux atlas and fornix (seeding thalamus also seeds some parts of fornix). gFA of the fornix tract bundles by itself are not increased in the IGE patients compared to controls. Therefore, the

abnormal increase in tract integrity of IGE patients is driven by the superior-inferior cortico-thalamic tracts shown in blue in Supplementary Figure 1 panel (h)-(k).

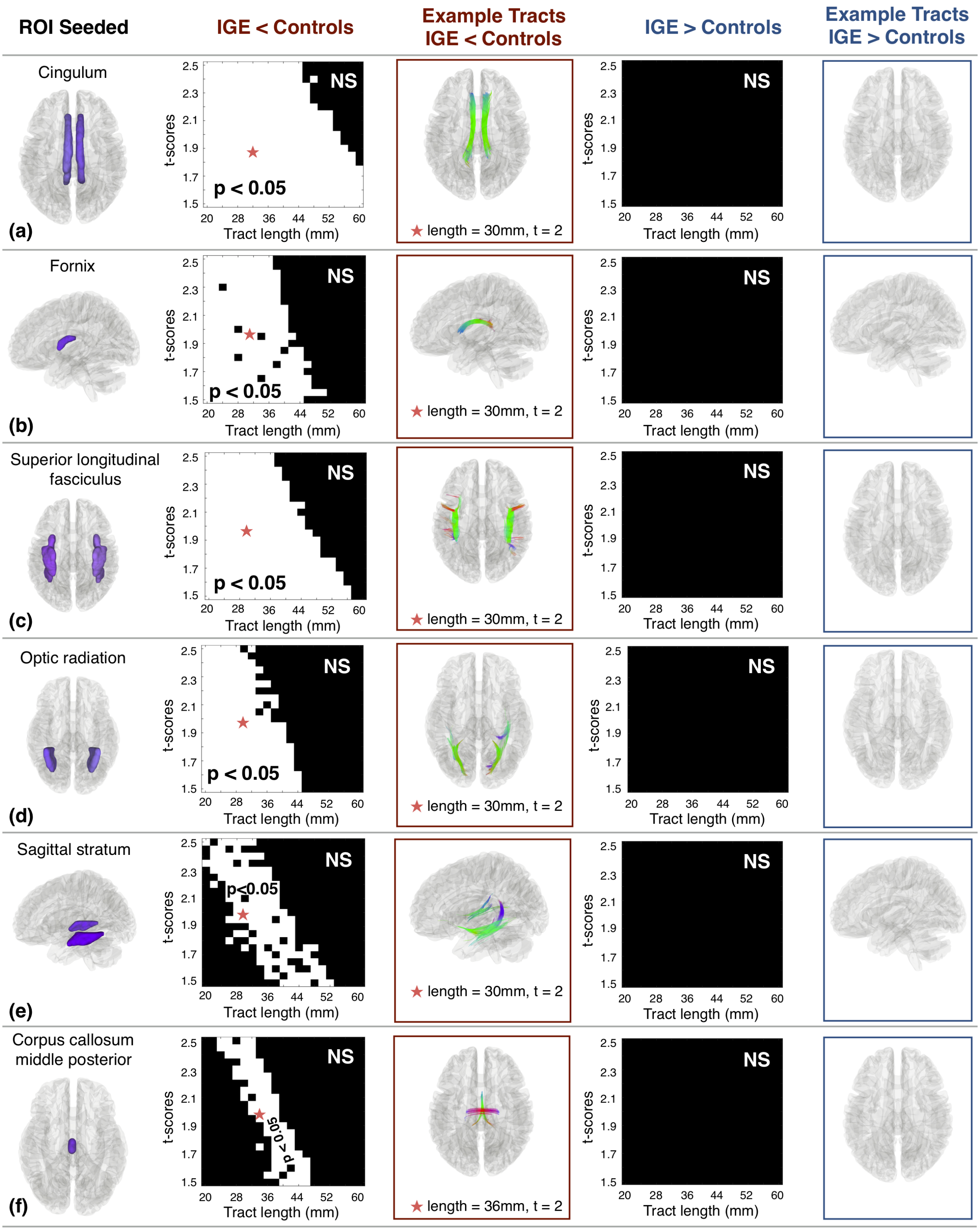


**Supplementary Figure 1: Connectometry analysis upon seeding pre-defined regions of interest** *(part 1)* Cortico-cortical tracts have significantly reduced structural integrity in IGE patients compared to controls for ROIs shown in panel (a)-(f). Tracts with reduced gFA are shown against each ROI for exemplary threshold value mentioned within the red box and illustrated by a red star. Note that for the ROIs seeded in panel (a)-(f), we detect no tract bundles with significantly increased gFA in IGE patients compared to controls. Colour coding of the tracts indicate direction as follows: red, left-right; green, anterior-posterior; blue, superior-inferior.

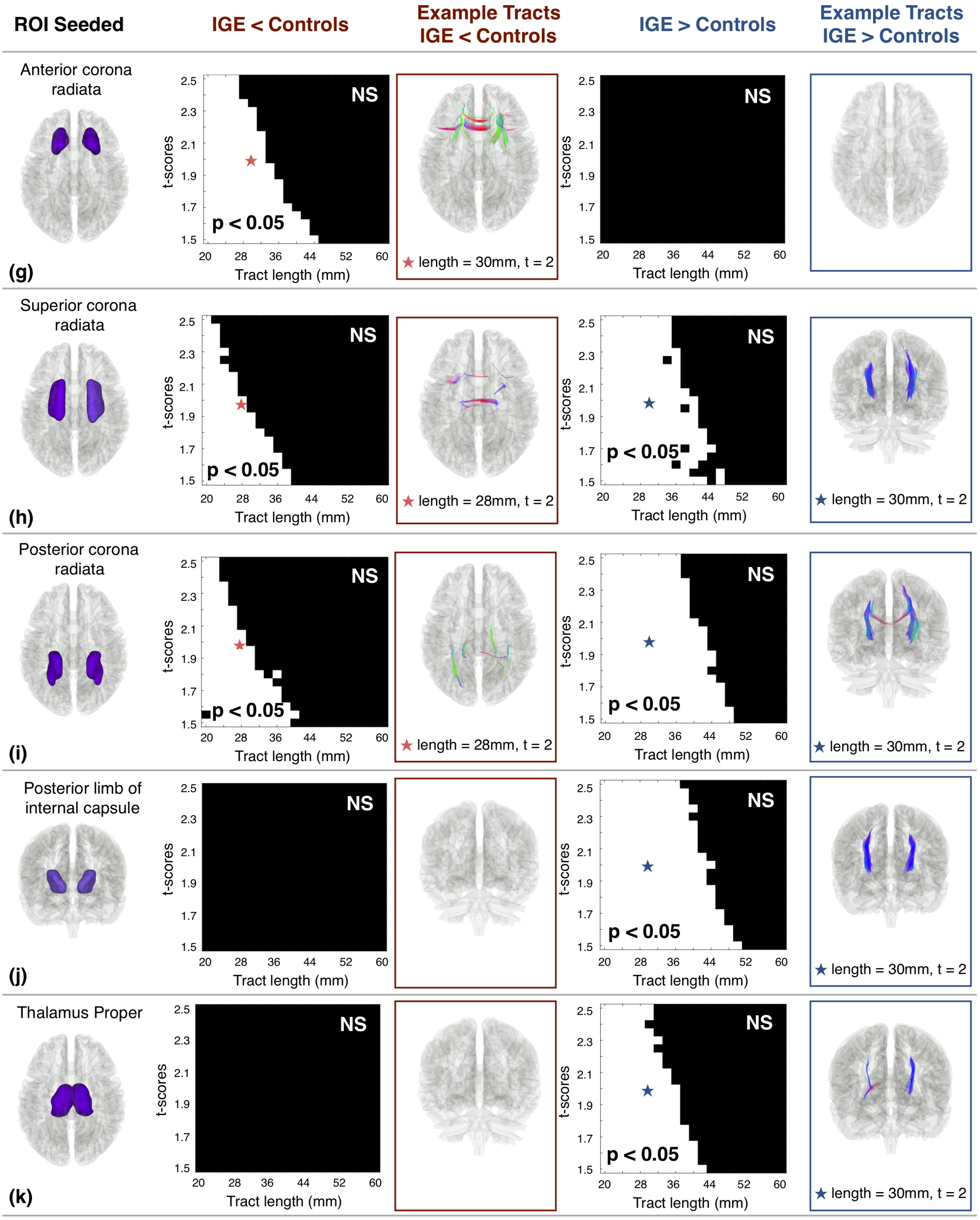


**Supplementary Figure 1: Connectometry analysis upon seeding pre-defined regions of interest** ***(part 2)*** gFA of cortico-cortical tracts were significantly reduced in IGE patients compared to controls for the ROIs seeded in panel (g)-(i). Additionally, cortico-thalamic tracts were found to be increased in IGE patients compared to controls upon seeding the ROIs in panel (h), (i) and (j). Tracts with reduced (increased) gFA are shown against each ROI for an exemplary threshold value mentioned within the red (blue) box and illustrated by a red (blue) star. In panel (k), thalamus was seeded to confirm that the cortico-thalamic tracts are indeed significantly increased in IGE patients compared to controls. Colour coding of the tracts indicate direction as follows: red, left-right; green, anterior-posterior; blue, superior-inferior.

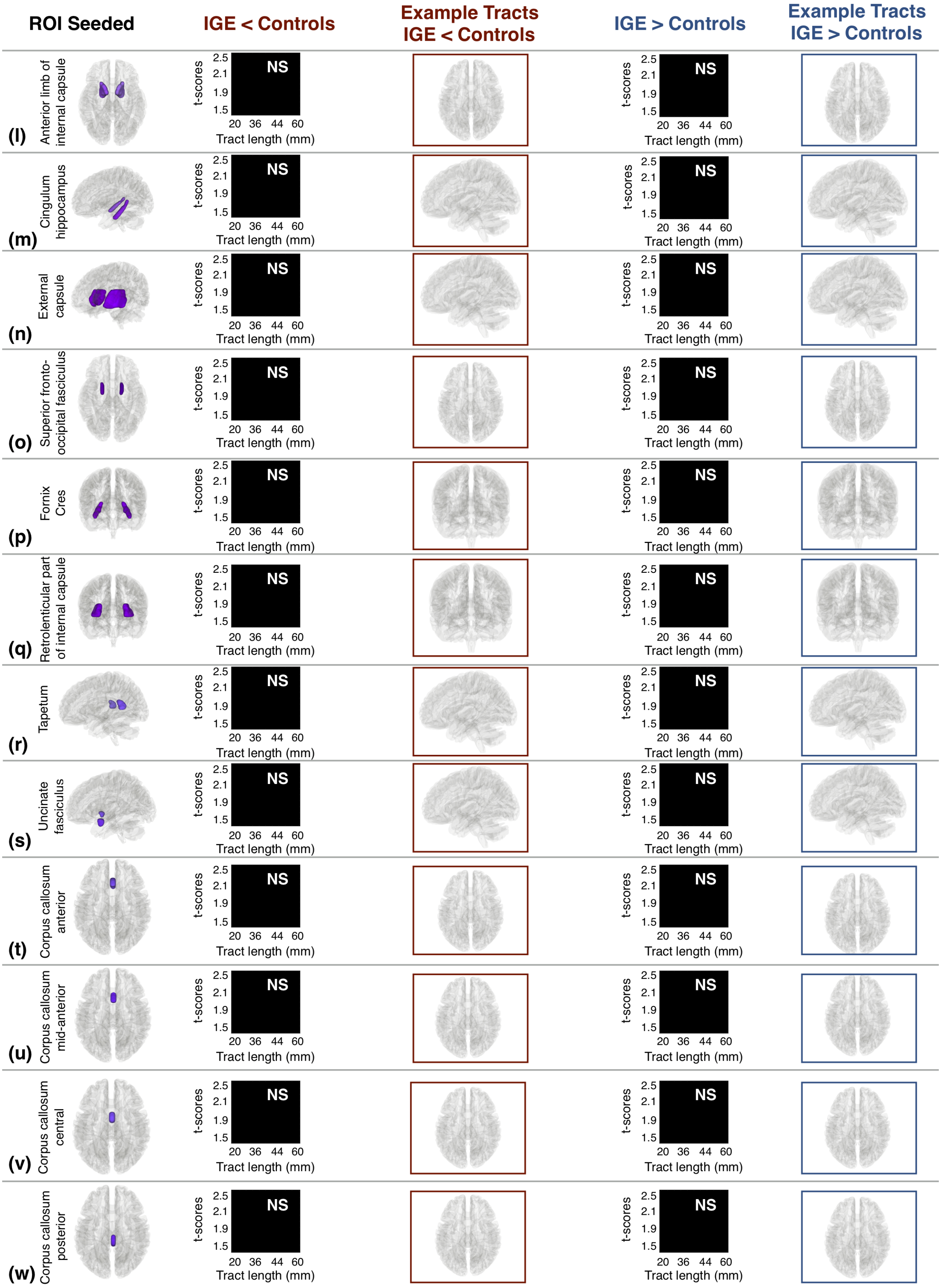


**Supplementary Figure 1: Connectometry analysis upon seeding pre-defined regions of interest** *(part 3)* No significant increase or decrease in gFA was detected between IGE patients and controls for any tracts upon seeding the ROIs shown in panel (l)-(w).

### 1.2. Connectometry analysis: Seeding whole brain

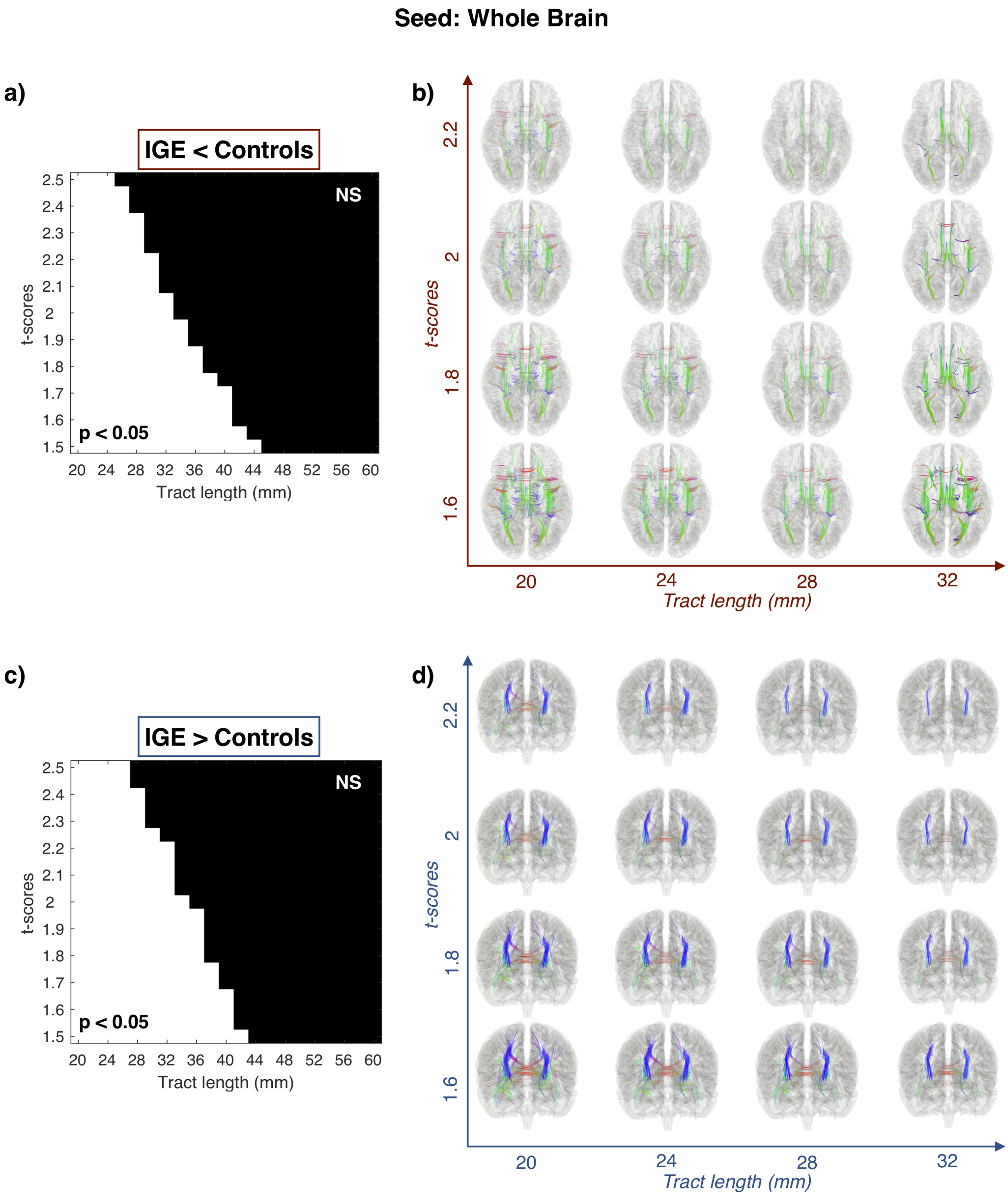


**Supplementary Figure 2: Connectometry analysis upon seeding the whole brain.** We seeded the whole brain in connectometry analysis to visualise the tracts with increased/decreased gFA between IGE patients and controls. As depicted by the white region in panel **(a)** and **(c)**, there indeed are tracts with significantly enhanced and reduced integrity. These tracts are visualised at exemplary pair of threshold values in panel **(b)** an **(d)**. Even though visually it may be difficult to attribute the specific tract bundles that may be driving the increases and decreases of mean gFA between patients and controls, consistent patterns are quite apparent. For example, at thresholds ($t = 2.2$, $l = 32mm$) in panel **(b)** cingulum, fornix, superior longitudinal fasciculus, and optic radiations are reduced, whereas for the same point in panel **(d)** cortico-thamic tracts are increased in IGE patients. These changes can, however, be quantified region specifically upon seeding the individual ROIs (c.f., Supplementary Figure 1). Colour coding of the tracts indicate direction as follows: red, left-right; green, anterior-posterior; blue, superior-inferior.

*1.3. Thalamo-frontal white-matter integrity is preserved in IGE patients*

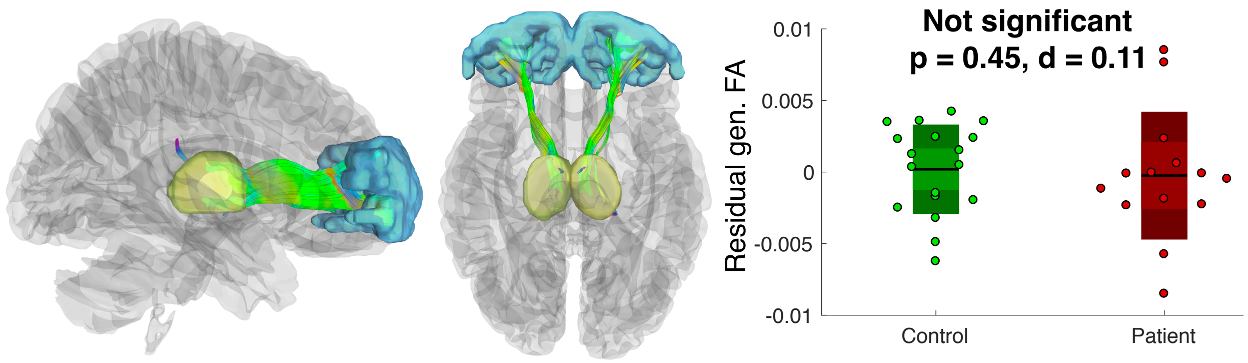


**Supplementary Figure 3: Thalamo-frontal white-matter integrity is preserved in IGE patients.** Confirming the results in McGill et al. 2014, we also find that the thalamo-frontal white-matter connectivity is preserved in patients with IGE. End to end tracking was performed between thalamus and medial prefrontal cortex ROIs. The thalamo-frontal tracts delineated and the ROIs are shown on the brain schematic. Mean gFA residuals for voxels underlying the delineated thalamo-frontal tracts were compared between the patients and control groups. No significant difference was found in the delineated thalamo-frontal tract integrity. Colour coding of the tracts indicate direction as follows: red, left-right; green, anterior-posterior; blue, superior-inferior.

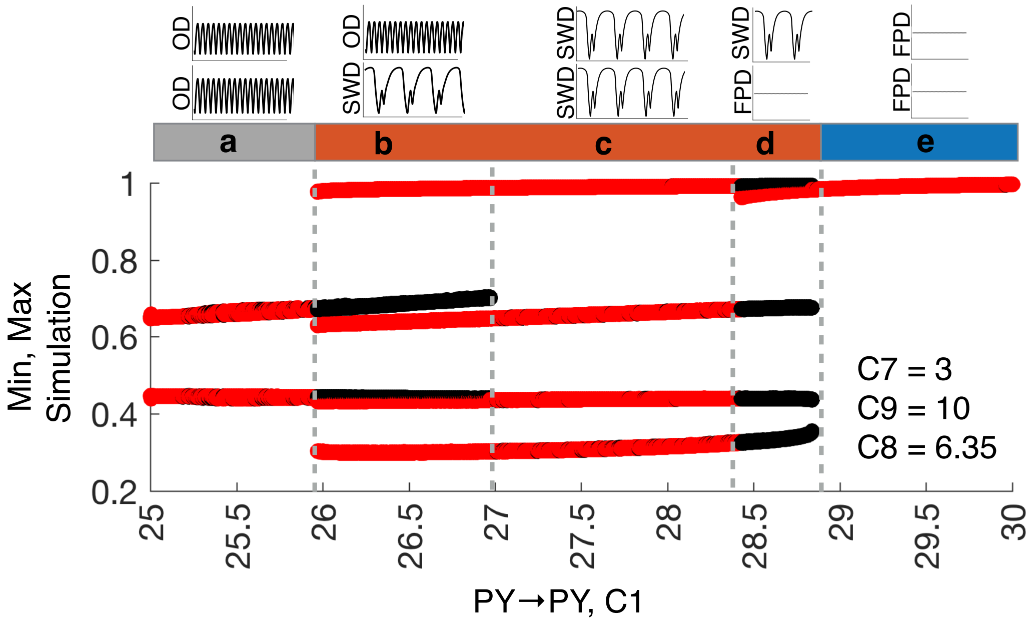


**Supplementary Figure 4: Bifurcation diagram illustrating detailed model dynamics.** The fixed point dynamics (FPD) representing seizure free activity is shown in blue in panel **(e)**. The model is bistable in regions shown by panel **(d)**. This means that for those parameter conditions the dynamics can either be a fixed point or a spike-wave (SWD) - i.e. both states coexist. With parameter setting in panel **(c)**, the dynamics become monostable with only spike-wave. In panel **(b)**, the model captures a bistable spike-wave dynamics with fast oscillatory dynamics (OD). Finally, the model is also capable of capturing a monostable fast oscillatory dynamics shown in grey in panel **(a)**.

*1.5. Whole-tract approach obscures significant local differences in white matter*

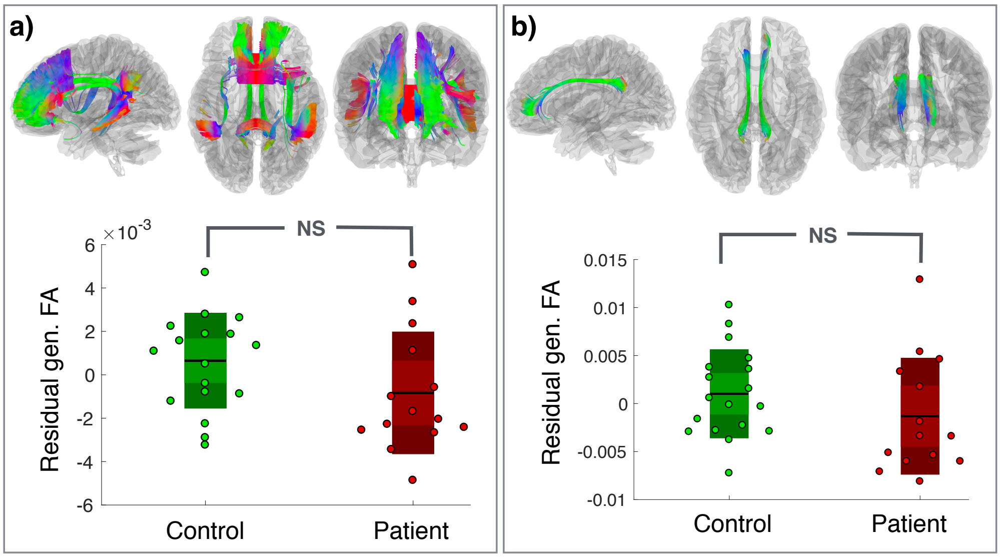


**Supplementary Figure 5: Whole-tract based approach obscures significant regional differences in white matter structures. a)** To delineate the white-matter tracts in the default mode network (DMN), we incorporated the DMN cortical parcellation scheme estimated by (Yeo et al. 2011) and performed the conventional end-to-end tractography. As evident by the box plot, the residuals of gFA averaged across the entire tract profile in the DMN is lower in IGE patients, but not significantly different than controls ($p = 0.12, d = 0.438$). **b)** Similarly, by conventional end-to-end fibre tracking the cingulum tracts connecting posterior cingulate cortex (PCC) with the medial prefrontal cortex (MPFC) are delineated. The box plot illustrates that the gFA residuals averaged across these tracts are reduced in IGE patients, but not significantly different in statistical sense from the control population ($p = 0.09, d = 0.592$). Colour coding of the tracts indicate direction as follows: red, left-right; green, anterior-posterior; blue, superior-inferior.

## 2. Supplementary Tables

### *2.1. Information on control subjects*

**Supplementary Table 1: Information on control subjects**

| Control | Gender | Age at MRI |
|---|---|---|
| 1 | Male | 40.2 |
| 2 | Female | 35.2 |
| 3 | Male | 45.1 |
| 4 | Female | 25 |
| 5 | Male | 33.1 |
| 6 | Female | 20.9 |
| 7 | Female | 23.7 |
| 8 | Male | 33.3 |
| 9 | Male | 22.8 |
| 10 | Female | 30.5 |
| 11 | Female | 28.9 |
| 12 | Male | 26.9 |
| 13 | Male | 22.3 |
| 14 | Female | 21.7 |
| 15 | Female | 37 |
| 16 | Female | 46.5 |
| 17 | Male | 28.9 |
| 18 | Male | 21.6 |

### *2.2. Values of the parameters incorporated in the model.*

**Supplementary Table 2: Values of the parameters incorporated in the model.**

| Parameter | Interpretation | Value |
|---|---|---|
| C1 | PY→PY connectivity strength | Variable |
| C2 | PY→IN connectivity strength | 38 |
| C3 | IN→PY connectivity strength | 25 |
| C4 | RE→RE connectivity strength | 0.1 |
| C5 | TC→RE connectivity strength | 2 |
| C6 | RE→TC connectivity strength | 1 |
| C7 | PY→TC connectivity strength | Variable |
| C8 | PY→RE connectivity strength | Variable |
| C9 | TC→PY connectivity strength | Variable |
| $\tau_1$ | PY timescale | 0.033 |
| $\tau_2$ | IN timescale | 0.013 |
| $\tau_3$ | TC timescale | 0.065 |
| $\tau_4$ | RE timesclae | 0.065 |
| $h_{py}$ | Input PY | -5 |
| $h_{in}$ | Input IN | -15 |
| $h_{tc}$ | Input TC | 10 |
| $h_{re}$ | Input RE | 5 |
| $\theta$ | Sigmoid threshold | 4 |
| $\alpha$ | Sigmoid steepness | 1 |

### References

McGill, M. L., Devinsky, O., Wang, X., Quinn, B. T., Pardoe, H., Carlson, C., Butler, T., Kuzniecky, R., Thesen, T., 2014. Functional neuroimaging abnormalities in idiopathic generalized epilepsy. YNICL 6, 455–462.

Yeo, B. T. T., Krienen, F. M., Sepulcre, J., Sabuncu, M. R., Lashkari, D., Hollinshead, M., Roffman, J. L., Smoller, J. W., Zollei, L., Polimeni, J. R., Fischl, B., Liu, H., Buckner, R. L., 2011. The organization of the human cerebral cortex estimated by intrinsic functional connectivity. Journal of Neurophysiology 106 (3), 1125–1165.
